# Multiwavelength tissue-mimicking phantoms with tunable vessel pulsation

**DOI:** 10.1117/1.JBO.28.4.045003

**Published:** 2023-04-17

**Authors:** Sophie Jenne, Hans Zappe

**Affiliations:** University of Freiburg, Department of Microsystems Engineering—IMTEK, Gisela and Erwin Sick Chair of Micro-Optics, Freiburg, Germany

**Keywords:** tissue phantom, skin equivalents, pulsation, polydimethylsiloxane, photoplethysmography, biomedical applications

## Abstract

**Significance:**

For the development and routine characterization of optical devices used in medicine, tissue-equivalent phantoms mimicking a broad spectrum of human skin properties are indispensable.

**Aim:**

Our work aims to develop a tissue-equivalent phantom suitable for photoplethysmography applications. The phantom includes the optical and mechanical properties of the three uppermost human skin layers (dermis, epidermis, and hypodermis, each containing different types of blood vessels) plus the ability to mimic pulsation.

**Approach:**

While the mechanical properties of the polydimethylsiloxane base material are adjusted by different mixing ratios of a base and curing agent, the optical properties are tuned by adding titanium dioxide particles, India ink, and synthetic melanin in different concentrations. The layered structure of the phantom is realized using a doctor blade technique, and blood vessels are fabricated using molding wires of different diameters. The tissue-mimicking phantom is then integrated into an artificial circulatory system employing piezo-actuated double diaphragm pumps for testing.

**Results:**

The optical and mechanical properties of human skin were successfully replicated. The diameter of the artificial blood vessels is linearly dependent on pump actuation, and the time-dependent expansion profile of real pulse forms were mimicked.

**Conclusions:**

A tissue equivalent phantom suitable for the *ex-vivo* testing of opto-medical devices was demonstrated.

## 1 Introduction

Tissue-mimicking phantoms facilitate the development and routine quality control of optical medical systems.^1^^,^^2^ Depending on the intended application, tissue-mimicking phantoms are designed to imitate optical, mechanical, and structural properties of human tissue,^3^^,^^4^ which restricts the materials that can be chosen for their fabrication.^5^ Tissue phantoms may be fabricated on the basis of either liquid or solid materials,^6^ but solid phantoms have advantages over their liquid counterparts including an increased long-term stability and signal-to-noise ratio, which are essential for the development of new biomedical measurement techniques.^7^^,^^8^ Furthermore, solid phantoms are suitable for the integration of different features of the tissue type to be mimicked, such as artificial bones, blood vessels, or even layered structures, representing different tissue layers.^7^

One important optical diagnostic technique that requires a multilayered design of suitable tissue phantoms with an embedded, expandable blood vasculature is photoplethysmography (PPG). PPG is a fast and noninvasive spectroscopic measurement technique^9^ used to detect changes of the blood volume in peripheral circulation^10^ either in reflection or transmission mode.^11^ The acquired spectra show a constant offset, i.e., a direct current (DC) component, which is caused by the absorption and scattering of the tissue under investigation and the constant blood volume inside the blood vessels. A time-variant alternating current component resulting from the increasing and decreasing blood volume during systole and diastole, corresponding to the desired measurement signal, is superimposed on this DC background.^11^^–^^13^ Using the PPG approach, a variety of clinical parameters can be determined, including the blood oxygen saturation, heart rate, and blood pressure.^14^^,^^15^ Furthermore, a PPG measurement is able to assess arterial diseases, as well as arterial compliance and aging,^16^^,^^17^ which makes it a highly useful tool in medical diagnostics.

Recently presented approaches for the fabrication of tissue-simulating phantoms that can assist in the development of PPG methodologies include the manufacturing of artificial blood vessels by molding metal wires^18^ or embedding of commercial^19^ or custom-fabricated tubing.^20^ In addition, Liu et al.^21^ demonstrated the integration of digital light process printed tissue layers containing 3D structured blood channels into a multilayer tissue phantom mimicking the epidermis, dermis, and hypodermis. Nevertheless, the tissue phantoms presented to date do not meet one or more of the requirements for realistic PPG-suited tissue phantoms, which are:

1. multilayer design mimicking the three outermost human skin layers of epidermis, dermis, and hypodermis;
2. mimicking of tissue optical properties;
3. mimicking of tissue mechanical properties;
4. embedding of all blood vessels that affect the PPG signal; and
5. quantified expansion of the artificial blood vessels during systole and diastole of a heartbeat.

The tissue phantom presented here fulfills all of the abovementioned requirements. It was fabricated from polydimethylsiloxane (PDMS) with tunable mechanical properties, and the optical properties were adjusted by adding India ink, titanium dioxide (${\mathrm{TiO}}_{2}$) particles, and synthetic melanin. The individual tissue layers were fabricated using the doctor blade technique, and the blood vessels were fabricated using molding and subsequent removal of wires of different diameters at different depths. Finally, using an artificial circulatory system, the expansion of the artificial blood vessels was monitored, and the pulsatile features of the tissue phantom were characterized.

## 2 Tissue Phantom Design and Fabrication

### 2.1 Design

**Fig. 1:**
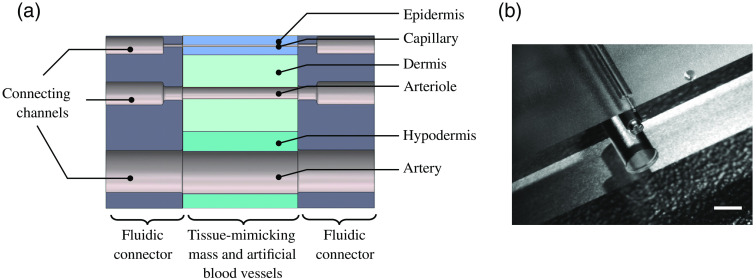
(a) Schematic sketch of the tissue phantom consisting of three different tissue-mimicking PDMS layers (green and blue, center) and a glass fluidic connector (gray, left and right). The channels mimic the blood vessels with a size (capillary, arteriole, and artery) corresponding to the particular skin layer. (b) Photograph of the fluidic connector that serves as part of the casting mold and fluidic interface for the tissue phantom, fabricated using SLE of glass. The scale bar is 600  μm.

As detailed in Fig. 1(a), the tissue-mimicking phantom primarily consists of two parts: (1) the multilayer PDMS tissue model itself and (2) a fluidic connector frame, which not only serves as a casting mold for the PDMS but also allows for clamping the wires used for the replication of the blood vessels. In addition, parts of the frame serve as a fluidic connector to integrate the tissue phantom into a circulatory system.

The multilayer phantom comprises (bottom to top) (1) the hypodermis with a thickness of $t_{1}=2\;\mathrm{mm}$ and embedded arteries with a diameter of $d_{1}=1000\;\mu m$, followed by (2) the dermis ($t_{2}=2\;\mathrm{mm}$) with arterioles ($d_{2}=200\;\mu m$) and the (3) epidermis ($t_{3}=500\;\mu m$) with capillaries ($d_{3}=50\;\mu m$) as the topmost layer.

The artificial tissue matrix is based on PDMS with tunable mechanical properties, as discussed in Sec. 4. In addition, absorbing and scattering agents were used to adapt the optical properties of the mixture, as described in Sec. 3.

### 2.2 Fabrication

**Fig. 2:**
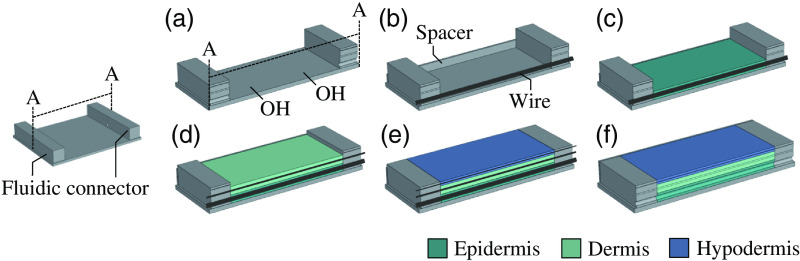
Schematic illustration of the fabrication process of the multilayer tissue phantom, which includes (a) the surface activation of the casting mold; (b) addition of a thickness spacer (corresponding to the thickness of particular skin layer) and wire (with a diameter corresponding to that of a particular blood vessel); (c) casting of the first tissue-mimicking layer of PDMS (epidermis); (d) second spacer, wire, and PDMS layer (dermis); (e) third spacer, wire, and PDMS layer (hypodermis); (f) removal of the wires to generate the blood-vessel channels.

The fabrication process for the multilayer tissue phantom with embedded artificial blood vessels consists of several individual fabrication steps, which are summarized in Fig. 2.

The mold frame was assembled from two fluidic connectors and spacers, as seen in Fig. 2. The fluidic connectors were fabricated in glass using selective laser-induced etching (SLE), a 3D structuring technique. In this process, the structure is laser written into fused silica (Toppan Inc.) using a laser microscanner (LightFab GmbH). Subsequently, the glass is etched in a potassium hydroxide solution with a concentration of 8  mol/L at a temperature of 78°C for 48 h under ultrasound irradiation, removing the laser-exposed volumes.

Spacers used to define the thicknesses of the three PDMS tissue layers were fabricated from a photopolymer resin (FLGPR04, Formlabs Inc.) using a stereolithographic printer (Form 3, Formlabs Inc.). The fluidic connectors and two spacers with a height of 2 mm were then installed on a glass plate (microscope slide, $75\times 50\;{\mathrm{mm}}^{2}$, Corning Inc.) using an ultraviolet (UV) curable epoxy resin.

On the glass slide, the fluidic connectors were placed parallel to each other, with a distance of 30 mm, and the spacers were placed in such a way that the casting mold was leak-tight. Subsequently, to remove organic contamination and activate polar surface groups, the glass surface was oxidized in an oxygen plasma for 90 s, as illustrated in Fig. 2(a).

Subsequently, as seen in Fig. 2(b), a wire with a diameter of 1000  μm (silver wire, BKL Electronic Kreimendahl GmbH) was clamped into the mold frame by pulling it through the bottom-most channels of the fluidic connectors and temporarily attaching it on the glass plate using tape (polyimide film silicone tape, Toolcraft, Conrad Electronic SE). To enhance the delamination of the wires from the PDMS, the wires first were coated with parylene-C.

After that, as shown in Fig. 2(c), the hypodermis-mimicking PDMS mixture was poured into the casting mold, and the excess material was removed using a blade. The mixture was then allowed to cure for 24 h at room temperature. The dermis and epidermis were fabricated in the same manner [Fig. 2(d) and 2(e)], using 200  μm and 50  μm wires (Enameled copper wire, TRU Components, Conrad Electronic SE and Stroft GTM, WAKU GmbH). After deposition of the epidermis-mimicking layer [Fig. 2(f)], everything was allowed to cure for an additional 48 h, and the wires were removed carefully, which finally led to the completed multilayer tissue phantom with hollow channels.

Using this technique, five samples of an artificial epidermis with a mean thickness of 487±19  μm were fabricated, whereas the dermis and hypodermis had a mean thickness of 1984±84  μm and 1854±45  μm, respectively. The mean diameter of the artificial capillaries, arterioles, and arteries was 59±4  μm, 217±11  μm, and 989±13  μm, respectively.

### 2.3 Setup of the Artificial Circulatory System

**Fig. 3:**
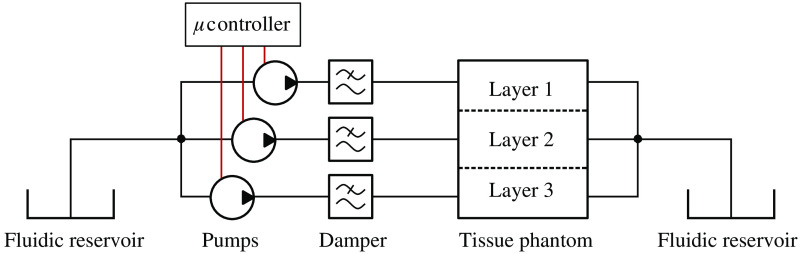
Illustration of the artificial circulatory system consisting of the tissue phantom and multiple fluidic elements: fluid reservoirs, microprocessor-controlled displacement pumps, and pulsation dampers. Suitable tubing interconnects the individual components, and the pumps are controlled via an evaluation board. Fluidic connections are displayed in black; electrical connections are in red.

For characterization of these phantoms, pulsatile pressure waves through the vessels were stimulated using three microprocessor-controlled displacement pumps (mp6-liq, Bartels Mikrotechnik), as depicted in Fig. 3.

Each of the miniaturized pumps actuated the artificial “blood” (i.e., deionized water in this case) through a different artificial tissue layer to allow for independent control of the liquid flow in each layer. For the experiments presented here, the operating frequency of the micropumps was kept constant at 100 Hz, and changes in the flow profile were only generated by varying the driving voltage of the pumps within a voltage range of 0 to $250\;V_{\mathrm{pp}}$ (see Sec. 5.1 below).

Dampers (mp-damper, Bartels Mikrotechnik) were placed in between the pumps and the tissue phantom to reduce unwanted high-frequency pulsations in the flow generated by the micropumps. The pumping liquid was stored in two reservoirs located at the beginning and end of the fluidic circuit.

All fluidic components were interconnected using Tygon^®^ tubing with an inner diameter of 1.3 mm and Shore A hardness of 55, which is significantly higher than the hardness of the artificial vessels. Therefore, the expansion of the tubing is negligible when applying different driving voltages to the pumps. The tubing was connected to the tissue phantom using a combination of Luer-lock adapters (neoLab Migge GmbH) and 45° angled dispensing tips with different tip diameters (all from Vieweg GmbH). The fluidic interface was finally sealed with a UV curable epoxy resin (Vitralit 1605, Panacol-Elosol GmbH).

## 3 Modification of Optical Properties

### 3.1 Sample Preparation

The manipulation of the optical properties of the mold mass was achieved by adding black ink (Staedler) and synthetic melanin (Thermo Scientific) as absorbers and titanium dioxide powder (Merck) as the scattering agent in different concentrations to the PDMS. Commonly, tissue phantoms are fabricated using only one absorbing and one scattering agent. However, in this paper, we additionally used synthetic melanin to mimic the wavelength dependency of the absorption coefficient. This is possible because the absorption coefficient of the synthetic melanin decreases as the wavelength increases, as has also been observed in human tissue.^22^ Therefore, tissue phantoms fabricated using synthetic melanin are suitable for use in the development of multiwavelength PPG applications, a decided advantage of the device presented here.

The mixing protocol for the mixtures required for the different tissue layers consisted of several steps. To reduce concentration deviations when preparing multiple phantoms, stock solutions of the synthetic melanin powder and ${\mathrm{TiO}}_{2}$ powder with a particle size of <3  μm were prepared. Therefore, melanin and ${\mathrm{TiO}}_{2}$ were diluted in isopropanol, defined amounts of each stock solution were pipetted into a beaker, and the isopropanol was allowed to evaporate. After the powders were dried, the PDMS base material was added by weight. In parallel to that, the ink was pipetted into a second beaker containing the PDMS curing agent. Both mixtures were homogenized separately using a dispersing instrument (Ultra-Turrax, IKA-Werke GmbH). Then, both mixtures were mixed together, again homogenized for 2 min, and degassed for 15 min. Subsequently, the mixture was poured into the casting mold, and air bubbles were removed within three degassing cycles of 15 min each followed by a curing time of 48 h. For the investigation of the influence of each of the agents on the optical properties, samples containing only one of them were fabricated.

### 3.2 Determination of $\mu_{a}$ and ${\mu_{s}}^{\prime }$

The experimental data basis for the calculation of the optical properties of the different PDMS mixtures was generated by spectroscopic measurements. An ultraviolet-visible near-infrared spectrophotometer (UV-3600i Plus, Shimadzu Co.) including an integrating sphere (ISR1503, Shimadzu Co.) with a diameter of 150 mm was employed to measure the total reflectance and transmittance and the direct transmittance of all samples. The spectra were acquired in 1-nm steps from 500 to 1000 nm. In addition, the refractive indices of the samples were measured at 20°C using a multiwavelength refractometer (Schmidt+Haensch GmbH Co.) at nine different wavelengths.

The data were subsequently fitted within OpticStudio (Zemax LLC.) using the Sellmeier equation, and the dispersion formula coefficients were computed. In combination with the sample thickness, which was measured using a caliper, these values served as input values for the inverse adding doubling algorithm developed by Prahl.^23^ This model ultimately yielded values for $\mu_{a}$, $\mu_{s}^{\prime }$ from the experimental data basis.

### 3.3 Results

**Fig. 4:**
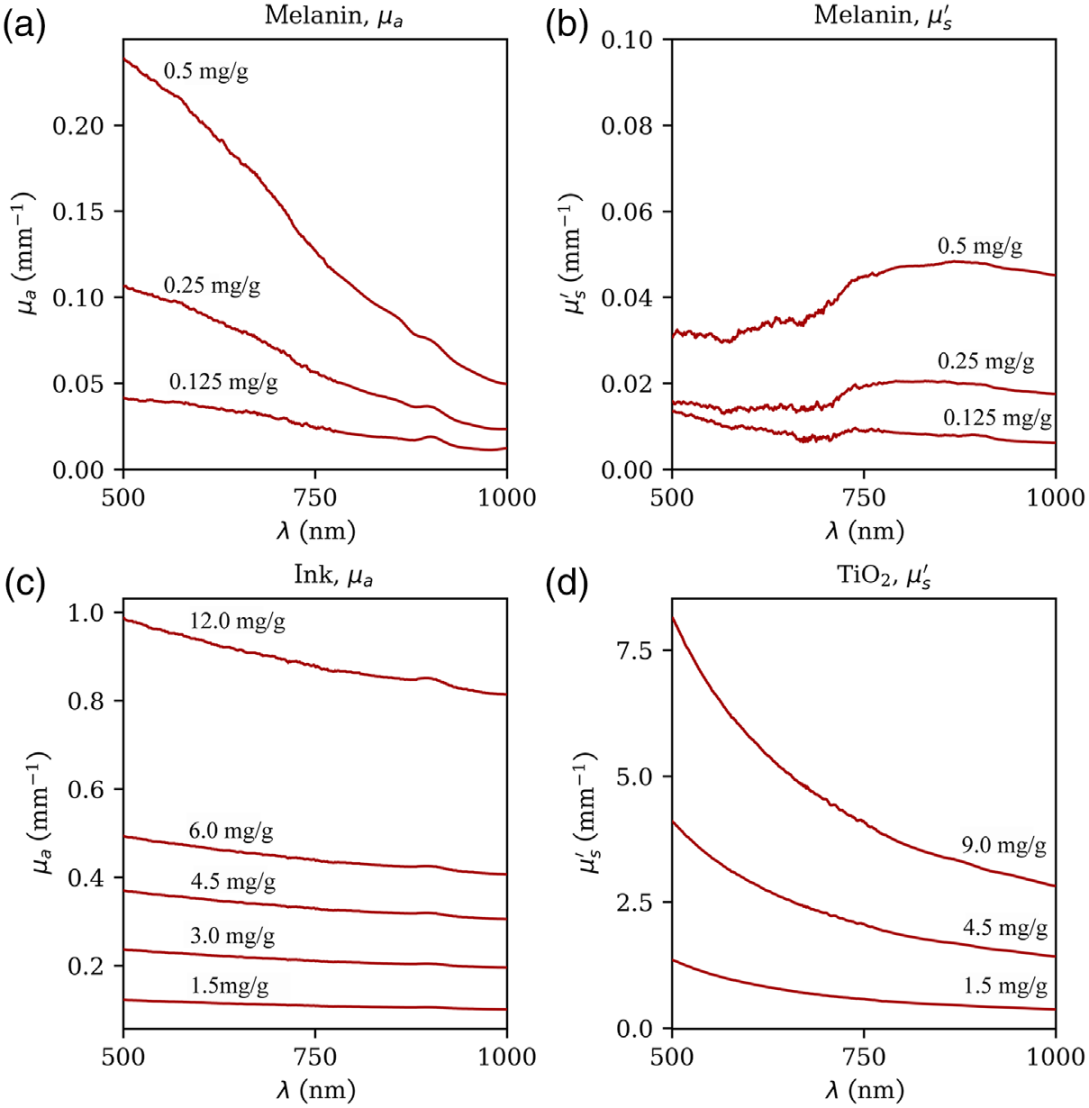
Measured absorption ($\mu_{a}$) and scattering ($\mu_{s}$) as a function of the concentration of melanin (a, b); ink (c); and ${\mathrm{TiO}}_{2}$ (d). Measurements were performed on samples containing only one of the dopants. Synthetic melanin alters both (a) absorption and (b) scattering, whereas (c) ink only changes the absorption properties and (d) ${\mathrm{TiO}}_{2}$ only changes the scattering properties.

The effects of melanin as the only dopant in PDMS on the absorption and scattering properties as a function of wavelength can be seen in Figs. 4(a) and 4(b). At 500 nm, the absorption was higher compared with longer wavelengths, which resembles the absorption behavior of human skin. Because synthetic melanin is a powder, it also effects the scattering. However, the scattering of the melanin concentrations needed for realistic phantom fabrication was significantly lower when compared with the scattering of ${\mathrm{TiO}}_{2}$ particles. As seen in Fig. 4(d), scattering increased as the wavelength decreased.

**Fig. 5:**
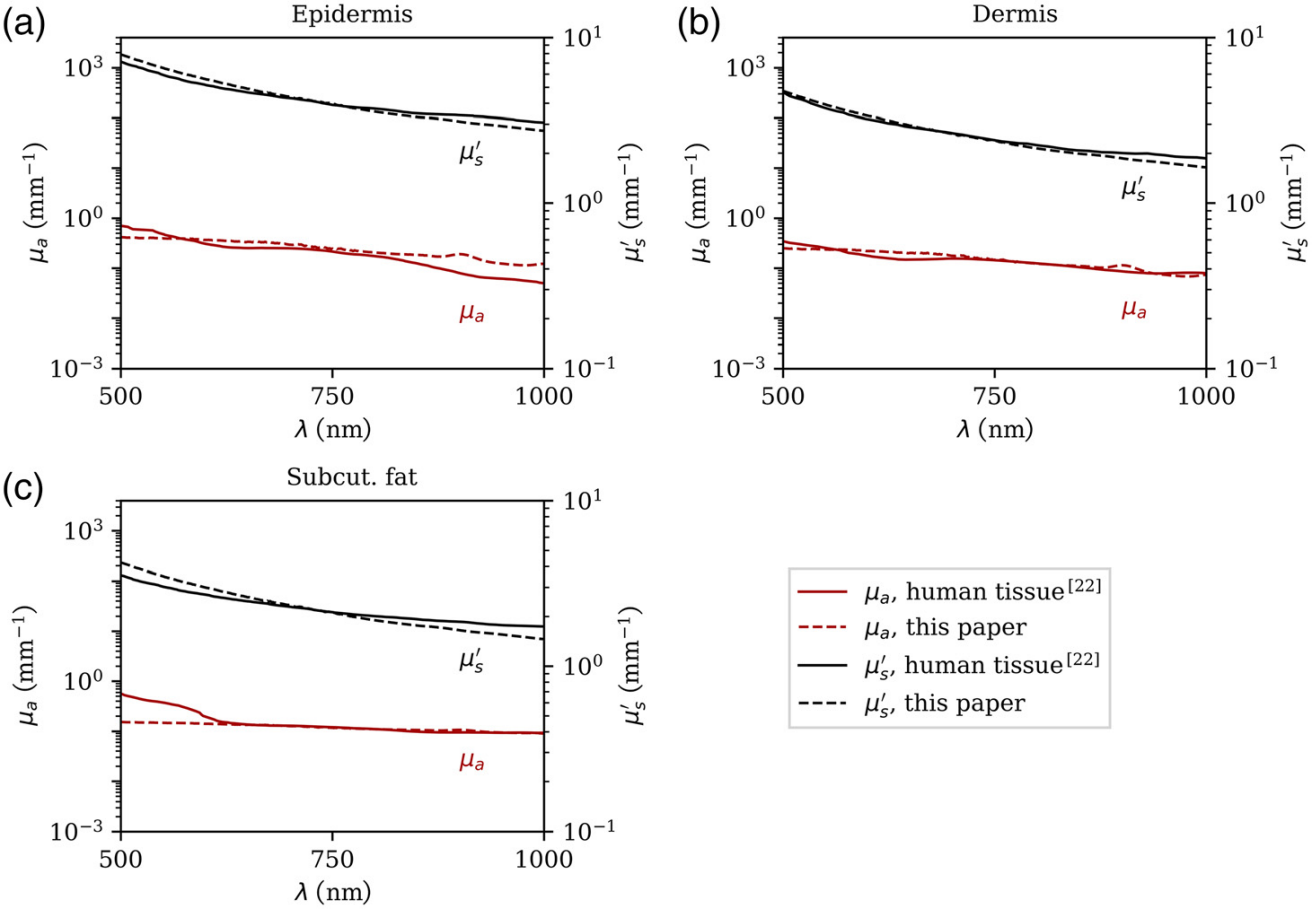
Comparison of the absorption ($\mu_{a}$) and scattering ($\mu_{s}$) coefficients of the (a) epidermis, (b) dermis, and (c) subcutaneous fat mold mixtures with tissue optical properties presented in the literature.^22^ The mixtures were prepared as shown in Table 1.

The wavelength dependence of the absorption coefficient of ink was found to be lower than that of melanin, as seen in Fig. 4(c), which makes it a suitable absorber in combination with synthetic melanin. Thus, a combination of ink, ${\mathrm{TiO}}_{2}$ powder and synthetic melanin allows for the fabrication of a single mold mass with realistic optical properties at multiple wavelengths. A possible combination of these materials to mimic the optical properties of the uppermost three skin layers in the range of 500 to 1000 nm is given in Table 1. Absorption and scattering occur more strongly in the epidermis compared with the other layers, which thus requires higher concentrations of scattering and absorbing agents. Figure 5 shows a comparison of the optical properties of human tissue reported in literature^22^ and the measured spectra of the tissue-mimicking mold mixtures prepared as summarized in Table 1. All three tissue types were mimicked realistically over the wavelength range under investigation.

**Table 1:** Summary of the concentrations of scattering and absorbing agents needed to mimic the optical properties of tissue^22^ in the range of 500 to 1000 nm. The concentration is given as the amount needed per gram of plain PDMS.

Tissue layer	$c_{\text{Melanin}}$	$c_{\text{Ink}}$	$c_{{\mathrm{TiO}}_{2}}$
	mg/g	mg/g	mg/g
Epidermis	1.25	0.00	8.55
Dermis	0.75	0.00	5.13
Subcutaneous fat	0.187	1.08	4.62

## 4 Modification of Mechanical Properties

### 4.1 Sample Preparation

The mechanical properties of the PDMS (Sylgard 184, Dow Corning) mixture were modified using different mixing ratios of the base material and the cross-linker, i.e., the curing agent. The weight ratios investigated were 1:10, 0.75:10, 0.5:10, and 0.25:10 (curing agent: base material) and the samples consisted of plain PDMS and PDMS mixed with the scattering and absorbing materials.

The mixing of the components was performed as described in Sec. 3.1, and the mixture was subsequently cast into custom-made casting molds and cured at room temperature. These molds were 3D printed (Prusa i3 MK3, Prusa Research s.a.) using polylactic acid. The geometries used for these molds were chosen in accordance with the ISO 527-2 standard, with a gauge length of 50 mm.

To enhance the delamination of the cured PDMS mixtures from the casting mold, the latter was surface-modified before casting using a soap-isopropanol mixture (mixing ratio 1:10) that was poured onto the casting mold and then allowed to rest until the isopropanol was fully evaporated and the casting mold surface was covered with a thin soap layer. The mechanical characterization was performed 5 days after the sample preparation.

### 4.2 Tensile Testing Procedure

To better understand the mechanical properties of the PDMS, uniaxial tensile testing was performed on samples using a universal testing machine (Inspekt Table 5, Hegewald & Peschke MPT GmbH) with a 1-kN load cell and a testing speed of 250  mm/min. Each sample was measured five times. Subsequently, the Young’s modulus was obtained from Hooke’s law by calculating the slope of the stress–strain curve in the linear-elastic region (i.e., 0%≤ϵ≤5%) using a linear fit within OriginPro 2022b (OriginLab Corp.).

### 4.3 Results

**Fig. 6:**
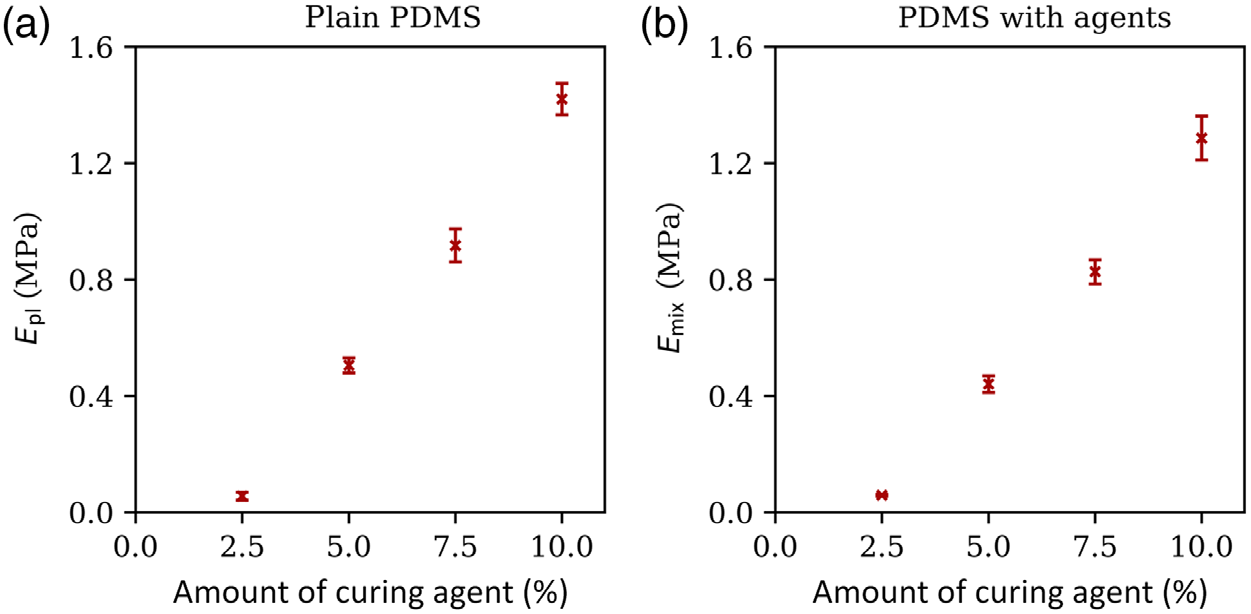
Young’s modulus measured for samples with different amounts of curing agent added to (a) plain PDMS and (b) PDMS mixed with scattering and absorbing agents. The concentrations of the optical scattering agents were chosen to be the highest amount that will be added to the plain PDMS, i.e., for the epidermis. The exact values can be extracted from 1. Young’s modulus increases as the amount of curing agent increases and is higher for plain PDMS compared with PDMS that was mixed with agents before curing. Data presented are mean ± standard deviation.

The Young’s modulus measured for plain PDMS samples and samples with scattering and absorbing agents is shown in Fig. 6. The Young’s modulus of the test samples was found to be linearly dependent on the amount of curing initiator used for sample preparation. For the plain PDMS samples, the Young’s modulus increased from 0.05  MPa±0.01  MPa to 1.42  MPa±0.05  MPa as the amount of curing agent was increased from 2.5% to 10.0% and from 0.05  MPa±0.01  MPa to 1.28  MPa±0.07  MPa for PDMS with scattering and absorbing agents.

The data are best fit by a linear regression, in which the addition of more curing agent causes a linear increase. The equation for the Young’s modulus of plain PDMS is $E_{\mathrm{pl}}=0.18\;\mathrm{MPa}/\%\cdot c-0.39\;\mathrm{MPa}$ ($R^{2}=0.99$) and $E_{\mathrm{mix}}=0.16\;\mathrm{MPa}/\%\cdot c-0.33\;\mathrm{MPa}$ ($R^{2}=0.99$), where c is the concentration of the curing agent (in %). This data show that, by adjusting the PDMS formulation, the resulting stiffness can be tuned to match the different mechanical properties of different tissue types.

Table 2 summarizes the PDMS formulation needed to mimic the Young’s modulus of the capillaries, arterioles, and arteries in the forearm. Arterioles have the lowest elastic modulus (0.12 MPa), which results in a curing agent concentration of 2.3% for plain PDMS and 2.8% for PDMS with scattering and absorbing agents added, whereas arteries show the highest elastic modulus, which results in curing agent concentrations of 7.7% (plain PDMS) and 8.3% (PDMS mixture), respectively.

**Table 2:** Amount of curing agent (in %) needed to mimic Young’s modulus (E in MPa) of different vessel types.

Vessel type	Artery	Arteriole	Capillary
E	1^19^	0.12^24^	0.37^24^
c, plain PDMS	7.7%	2.3%	4.2%
c, PDMS with agents	8.3%	2.8%	4.3%

## 5 Expansion of Channels

### 5.1 Fabrication of Phantom Equivalent

To allow for *in-situ* recording of the vessel expansion using a microscope (Zeiss Axioplan 2), phantom equivalents needed to be produced from plain PDMS without added scattering and absorbing agents. Those equivalents were fabricated in such a way as to have the identical mechanical properties as the phantom mixtures that will be ultimately used.

### 5.2 Calibration of Channel Expansion

The channel expansion was evaluated for three different tissue phantoms by recording photographs of the channels while increasing the pumping voltage from 0 to $250\;V_{\mathrm{pp}}$ in steps of 10 V. Subsequently, the photographs were evaluated within a Python routine. Herein, the acquired images were first converted into gray scale. Then, the channel diameters were calculated for each image from the gray value profile of a line of pixels perpendicular to the channel.

**Fig. 7:**
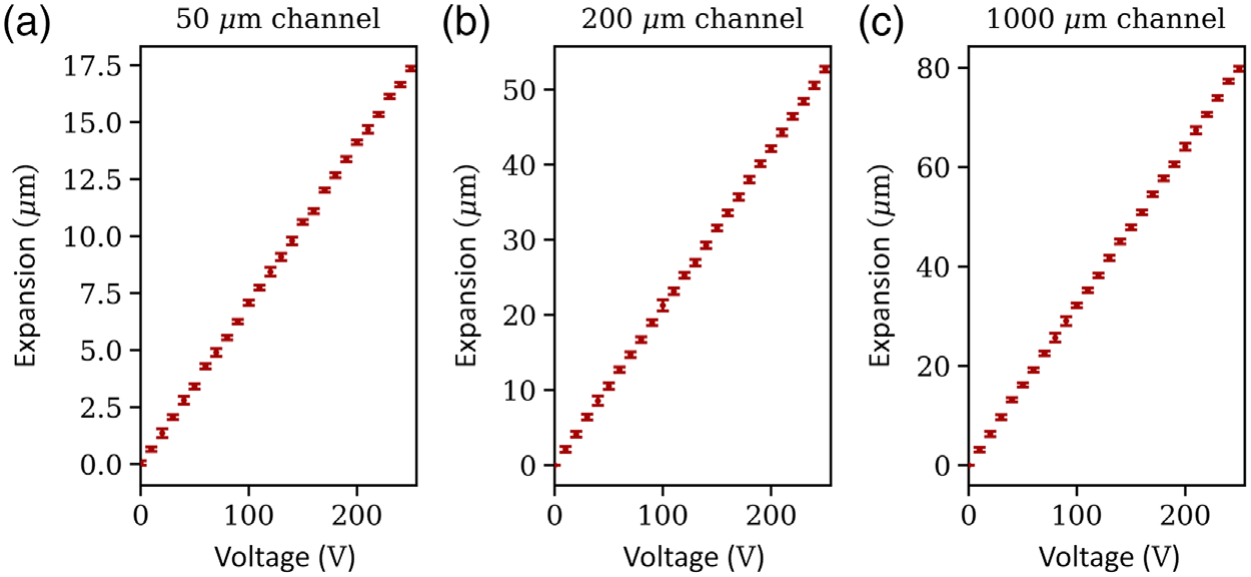
Radial expansion of artificial blood vessels with different diameters as a function of the pump actuation voltage: (a) 50-μm channel, (b) 200-μm channel, and (c)1000-μm channel. As the actuation voltage increases, the flow rate increases and the channel walls expand.

Figure 7 shows the radial expansion of the walls of channels with different diameters as a function of pump actuation. The relative displacement of the arteriole and capillary mimicking channels is higher compared with the larger diameter channel mimicking the artery. To explain this behavior, two counteracting effects need to be considered: (1) the PDMS used in the lowest layer, where the artery is located, is designed to have a higher Young’s modulus, which means that higher forces are needed to deform the channel walls. However, assuming a steady flow, constant density of the fluid, and no friction, (2) the static pressure in the channel decreases because the fluid velocity decreases as the cross-sectional area of the channels decreases according to Bernoulli’s equation. When comparing the different diameters of the channels, the highest pressure is present in the larger vessels; however, the expansion is larger in the smaller diameter vessels due to the lower Young’s modulus.

For all channel diameters under investigation, the channel expansion increased linearly as the pumping voltage increased. Hence, the voltage $V_{\text{pump}}$ that needs to be applied to the pumps to achieve a defined channel expansion u is calculated from the linear fit equations, given as

for $d_{1}=1000\;\mu m$:

$$u_{1}=(0.26\;\mu m/V)\cdot V_{\text{pump}},$$ (1)

for $d_{2}=200\;\mu m$:

$$u_{2}=(0.21\;\mu m/V)\cdot V_{\text{pump}},$$ (2)

and for $d_{3}=50\;\mu m$:

$$u_{3}=(0.07\;\mu m/V)\cdot V_{\text{pump}}.$$ (3)

### 5.3 Mimicking of Realistic Arterial Pulse

The channel expansion within a cardiac cycle was simulated based on the calibration model derived in Sec. 5.2. For the calculation of the radial arterial wall displacement Δu(t) caused by a pressure wave Δp(t), the artery was assumed to be a thick-walled cylinder consisting of an isotropic, single-layered wall. The external pressure impinging on the dilating vessel was assumed to be negligible because the Young’s modulus of the artery is three orders of magnitude higher than the Young’s modulus of the hypodermis tissue, which was determined to be 2 kPa by Pailler-Mattei et al.^25^

Using these assumptions, the equations for the differential radial and hoop stress, $\Delta \sigma_{r}$ and $\Delta \sigma_{\psi }$, respectively, and the plane-stress $\Delta \sigma_{z}$ present at the interior surface of the vessel, presented by Schesser et al.,^26^ simplify to

$$\Delta \sigma_{r}(t)=-\Delta p(t),$$ (4)

$$\Delta \sigma_{\psi }(t)=\Delta p(t)\cdot \frac{r_{i}^{2}+r_{o}^{2}}{r_{o}^{2}-r_{i}^{2}},$$ (5)

$$\Delta \sigma_{z}(t)=2\nu \Delta p(t)\cdot \frac{r_{i}^{2}}{r_{o}^{2}-r_{i}^{2}},$$ (6)

where $r_{i}$ and $r_{o}$ are the inner and outer vessel radius, respectively.

Furthermore, the radial strain is expressed by the kinematic relationship between the displacement and the extension as

$$\Delta \epsilon_{\psi }(t)=\frac{\Delta u(t)}{r_{i}}.$$ (7)

Assuming small strain, $\Delta \epsilon_{\psi }$ can also be expressed by the general form of Hooke’s law as

$$\Delta \epsilon_{\psi }(t)=\frac{1}{E}[\Delta \sigma_{\psi }(t)-\nu (\Delta \sigma_{r}(t)+\Delta \sigma_{z}(t))],$$ (8)

where E and ν are the Young’s modulus and Poisson’s ratio of the artery, respectively. Consequently, the radial displacement at the interior surface of the artery wall as a function of pressure change Δp(t) is finally derived as

$$u(t)=\frac{r_{i}\Delta p(t)}{E}[\frac{r_{i}^{2}+r_{o}^{2}}{r_{o}^{2}-r_{i}^{2}}+\nu (1-2\nu *\frac{r_{i}^{2}}{r_{o}^{2}-r_{i}^{2}})].$$ (9)

It is to be noted that the Young’s modulus of an artery is an incremental elastic modulus $E_{\mathrm{inc}}$ in practice,^27^ implying an increased elastic modulus as the wall displacement increases. However, we simplified our calculations by assuming it to be constant.

**Fig. 8:**
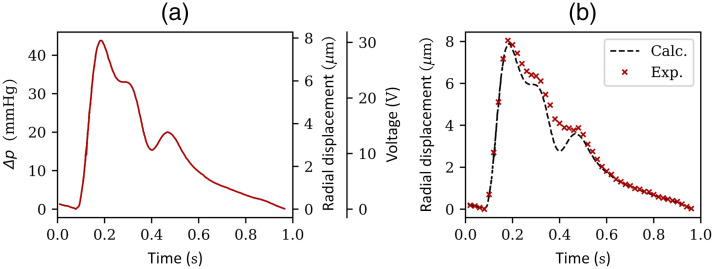
(a) From the known pressure pulse signal p(t) within one cardiac cycle (left scale), the expansion of the artery ($d_{1}=1000\;\mu m$) and the pump voltages needed to replicate this expansion can be derived (right scales). (b) The experimentally obtained radial displacement of the artery with a diameter of $d_{1}=1000\;\mu m$ using this voltage sequence is shown along with the calculated radial displacement.

Applying Eq. (9) to a known time-dependent form of a blood pressure pulse Δp(t)^28^ [Fig. 8(a), left scale] using an arterial wall thickness of 0.25 mm,^29^ the radial displacement of the vessel is calculated [Fig. 8(a), first right scale]. Then, using Eq. (1), the pumping voltages needed to expand the artificial blood vessels in the same manner as this cardiac pressure pulse are calculated [Fig. 8(a), second right scale]. The continuous voltage signal is converted to a discrete-time signal with 50 ms steps and used to operate the micro-pumps, which then ultimately results in the channel expansion shown in Fig. 8(b).

It can be clearly seen from Fig. 8 that the expansion of the channel using the designed custom pump sequence shows the expected behavior. The steep gradient of the systolic upstroke could be mimicked as expected. Furthermore, the minimal and maximal expansion of the vessel could be replicated at the point when the arterial valves open at $t_{1}\approx 0.1\;s$ and the maximum (systolic) pressure at $t_{2}\approx 0.19\;s$. However, higher deviation from the designed expansion shape can be observed for constricting channels, especially at the dicrotic notch at $t_{3}\approx 0.4\;s$, which can be explained by viscoelastic properties of the PDMS. However, it needs to be considered that real arteries at the dicrotic notch also do not contract as expected from this simplified calculation approach. Instead, the incremental Young’s modulus and the mechanical properties limit fast contractions.

## 6 Discussion and Conclusion

We have demonstrated a fabrication technique for multilayer tissue phantoms with pulsatile artificial blood vessels that can assist in the development of PPG applications. The phantom comprised the three upper-most skin layers, i.e., dermis, epidermis, and hypodermis, and the corresponding blood vessels capillaries, arterioles, and arteries. Our investigations showed that these tissue-simulating phantoms can be produced with high repeatability of the structural, mechanical, and optical properties.

With suitable absorbing and scattering agents, it is possible to tune the optical properties of a base material to match the optical properties of skin types with different melanin concentrations. Because we used synthetic melanin as an additional absorber, we achieved realistic skin optical properties and produced a phantom that is suitable for multiwavelength applications. The fabrication of tissue phantoms with a wide range of optical properties is especially important for overcoming the racial bias existing in pulse oximetry, which leads to an overestimation of the blood oxygen saturation of patients with a higher degree of skin pigmentation.^30^

Furthermore, we also quantified the change in the Young’s modulus of PDMS mixtures with different mixing ratios simulating tissue of different mechanical properties. We were able to tune the Young’s modulus within one order of magnitude by adjusting the amount of curing agent in the sample. The addition of the scattering and absorbing agent led to a slight decrease of the tensile stiffness compared with plain PDMS.

The optical modification of the PDMS mixture was stable over a monitoring period of 3 months. The mechanical stability of the tissue phantom was monitored over 2 weeks, and no changes could be observed. For both tests, samples were stored at room temperature.

In addition, we set up an artificial circulatory system and quantified the expansion of artificial blood vessels at different pump voltages. The expansion increased linearly with the pump voltage. A calibration model allows for the determination of the pump voltage needed to achieve a defined vessel expansion. Using the microprocessor-controlled pumps, we were therefore able to generate arbitrary pulse shapes with uniform expansion along the channel. Thus, we were able to significantly improve previously presented work.^31^

## Code, Data, and Materials Availability

The data that support the findings of this study are available upon reasonable request from the authors.
